# Strategies for developing self-assembled nanoparticle vaccines against SARS-CoV-2 infection

**DOI:** 10.3389/fimmu.2024.1392898

**Published:** 2024-09-11

**Authors:** Kaiwen Yang, Youqin Zeng, Xinyu Wu, Jia Li, Jinlin Guo

**Affiliations:** ^1^ College of Medical Technology, Chengdu University of Traditional Chinese Medicine, Chengdu, China; ^2^ Chongqing Key Laboratory of Sichuan-Chongqing Co-construction for Diagnosis and Treatment of Infectious Diseases Integrated Traditional Chinese and Western Medicine, Chongqing Traditional Chinese Medicine Hospital, Chongqing, China

**Keywords:** self-assembled nanoparticle, VLP, ferritin, SARS-CoV-2, vaccine

## Abstract

In the recent history of the SARS-CoV-2 outbreak, vaccines have been a crucial public health tool, playing a significant role in effectively preventing infections. However, improving the efficacy while minimizing side effects remains a major challenge. In recent years, there has been growing interest in nanoparticle-based delivery systems aimed at improving antigen delivery efficiency and immunogenicity. Among these, self-assembled nanoparticles with varying sizes, shapes, and surface properties have garnered considerable attention. This paper reviews the latest advancements in the design and development of SARS-CoV-2 vaccines utilizing self-assembled materials, highlighting their advantages in delivering viral immunogens. In addition, we briefly discuss strategies for designing a broad-spectrum universal vaccine, which provides insights and ideas for dealing with possible future infectious sarbecoviruses.

## 1 Introduction

Since the emergence of SARS-CoV-2, the rapid expansion and mutation of the virus have led to a significant increase in morbidity and mortality worldwide. According to the World Health Organization, the COVID-19 pandemic has resulted in more than 776 million confirmed cases, including nearly 7 million deaths (https://www.who.int/). During this period, vaccines have played a crucial role as a powerful health tool for preventing viral infection. The approval of multiple COVID-19 vaccines and vaccination campaigns in more than 200 countries have increased herd immunity, reduced disease severity and mortality, and helped curb the spread of pandemics (1). Current COVID-19 vaccines based on traditional vaccine platforms include CoronaVac, BBIBP-CorV, NVX-CoV2373, and Ad26.COV2-S, etc. (2). Studies from various countries indicate that the efficacy of the inactivated vaccines CoronaVac and BBIBP-CorV ranges from 50.7% to 91% (3). The protein subunit vaccine NVX-CoV2373 has shown an efficacy of 95.6% against the SARS-CoV-2 wild-type (WT) (4). Additionally, one dose of the viral vector vaccine Ad26.COV2-S was 67% effective against moderate disease and 77% effective against severe disease (5). Despite their high efficacy, these vaccine platforms have the weakness of limited immunogenicity, insufficient cross-protection, and the induction of anti-vector immunity (6, 7). These challenges have prompted the development and exploration of novel vaccine platforms that offer higher efficacy and fewer side effects.

The ongoing development of nanotechnology presents new opportunities for creating highly effective vaccines. Nanoparticle vaccines can be broadly categorized into two main types based on their strategies for delivering antigens (8). The first category encapsulates the antigen within the carrier, such as liposomal nanoparticles and polymeric nanoparticles (8). Among the approved COVID-19 vaccines, mRNA vaccines are impressive and stand out, as they encapsulate nucleic acid molecules of the antigen in a vector that delivers them to human host cells (9). The efficacy of mRNA vaccines, represented by BNT162b2 and mRNA-1273, can reach as high as 96.7% and 94.1% against severe disease, respectively (10, 11). The rapid development of mRNA vaccines, driven by revolutionary vaccine technologies, has set historic milestones and significantly advanced the clinical translation of nanoparticle vaccines. However, the widespread use of these vaccines is limited by storage stability (12). The second category of nanoparticle vaccines delivers the antigen to the surface of the carrier, which includes protein nanoparticles, virus-like particles (VLPs), and micelles (8, 13). Proteins with self-assembly capabilities are popular in this field. The varying copy numbers and geometries of self-assembling proteins enable diverse vaccine delivery options. Not only can antigens be densely arranged on their surfaces, but multivalent antigen presentation can also be achieved (14, 15). Additionally, cell-penetrating peptides and antigen-presenting cell (APC)-targeting antibodies can be incorporated onto the surface to enhance APC targeting and boost the immune response (16, 17). Furthermore, the strong thermal and chemical stability of certain self-assembled proteins helps overcome the challenges associated with cold chain transportation in underserved areas. Given these advantages, self-assembled nanoparticle (SANP) vaccines appear promising and will be highlighted and summarized in this review.

## 2 SANPs

**Figure 1:**
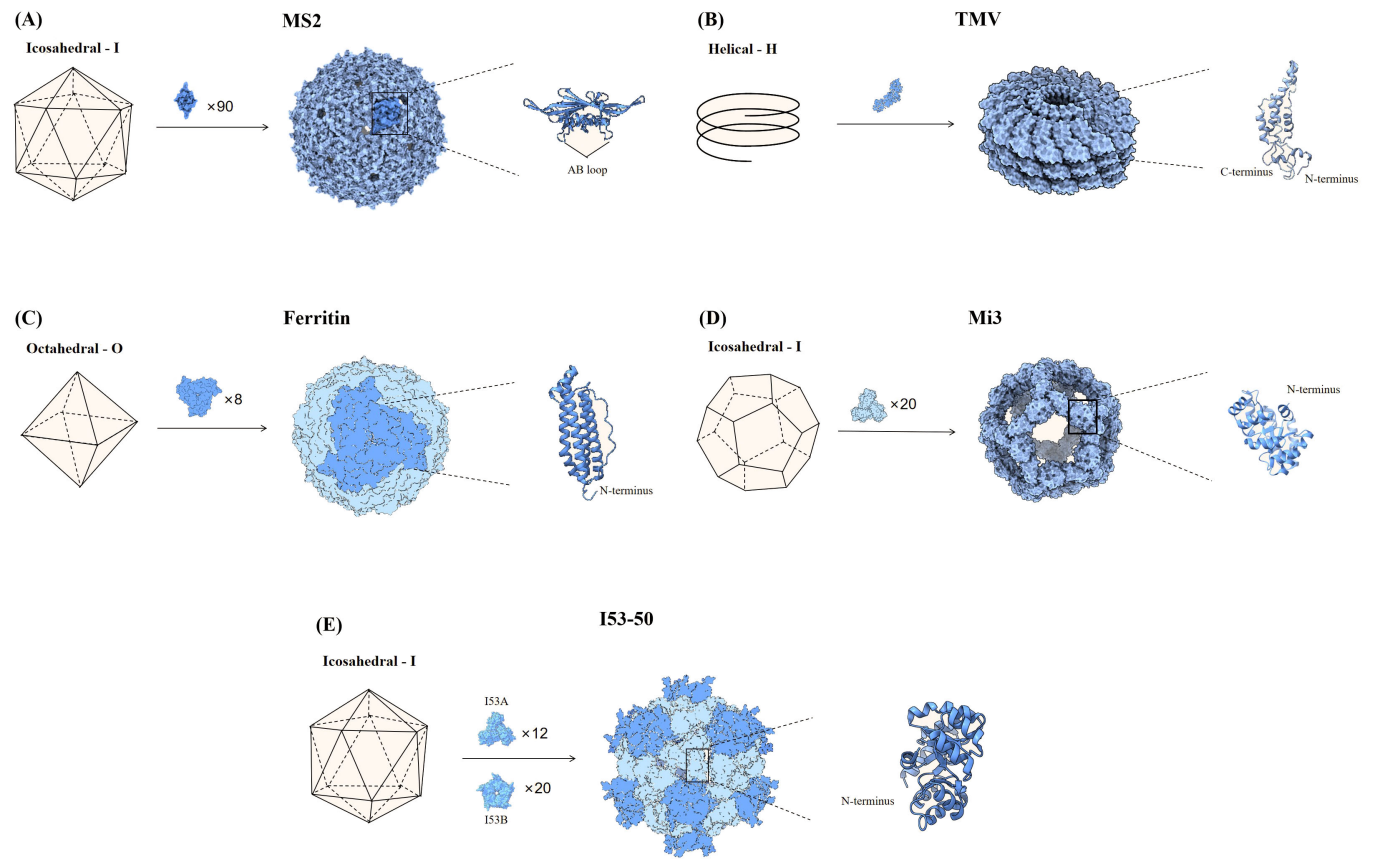
Partial nanoparticle structure and location of delivered antigens. **(A)** The MS2 phage consists of 90 dimers forming an icosahedron, and the AB loop of the homodimer is used for antigen presentation. **(B)** TMV is helical particle with the N- and C-terminus of the coat proteins exposed on the surface of the particle, which can be used to display antigens. **(C)** Ferritin consists of 8 trimers that form a sphere, and the N-terminus of each monomer is used to display antigenic. **(D)** Mi3 is shown as an icosahedron composed of 60 polymers, with the antigen attached to the N-terminus of each monomer. **(E)** I53-50 is an icosahedral nanosphere consisting of two parts, trimeric I53A and pentameric I53B. The N-terminus end of the monomeric I53A is connected to the antigen.

Generally, nanoparticles are tunable particles of nanoscale size that mimic the structural characteristics of viruses (8). When the attractive/repulsive forces within and between SANPs molecules are in equilibrium, the formation of various non-covalent interactions such as electrostatic, hydrophobic, hydrogen bonding, van der Waals, and π-π interactions allow the basic building blocks to assemble autonomously, a process that does not require human intervention (18, 19). Exogenous genes or antigenic proteins can be attached to specific positions in the basic building blocks of SANPs by gene fusion, tag coupling, etc., and antigens are presented to the surface of the particles as the basic building blocks of SANPs self-assemble (20) (**Table 1**) (**Figure 1**). Polysaccharides, ferritin, and other natural self-assembling molecules present in various organisms and the engineering self-assembling proteins have been widely used in the development of COVID-19 vaccines (**Table 2**).

**Table 1:** Regional pockets of individual nanoparticles to deliver antigens.

Nanoparticle	Protein	Region	Ref.
MS2	Coat protein homodimer	AB loop	(25)
TMV	Coat protein	N-terminus, C-terminus	(81)
PapMV	Coat protein	N-terminus	(82)
Ferritin	Ferritin	N-terminus	(48)
I53-50	I53A	N-terminus	(48)
Mi3	Engineered aldolase	N-terminus	(42, 45)

**Table 2:** Self-assembled nanoparticles in COVID-19 vaccine candidates.

Vaccine platform	Presentation method	Presented antigens	Sequence	Size	Route of administration	Immunization schedule	Animal models	Pseudovirus in neutralization assay	Live virus in neutralization assay	Fold of neutralizing potency compared to monomeric antigens	Th1 or Th2 oriented immune response	Ref.
MS2	Biotin-streptavidin	Spike	1–1208	~50 nm	Subcutaneous inoculation	1 dose (60 µg)	Hamster	——	SARS-CoV-2/UT-NCGM02/Human/2020/Tokyo	——	——	(26)
TMV	Chemically conjugated	RBD-Fc	331–632 from the WT	Diameter < 20 nm, average length 300 nm	Subcutaneous inoculation	2 doses (45 µg, 14 days apart)	Mice	WT	WT	10	Th1 (with adjuvants), balanced Th1 and Th2 response (without adjuvants)	(28)
PapMV	Sortase A	RBD	331–591 from the WT	Average length 80 nm, width 14 nm	Intramuscular injection	2 doses (115 μg of nanoparticles coupled to 4.6 μg of RBD, 21 days apart)	Mice	——	WT, Delta, and Omicron	54 (WT), 111 (Delta), 5.2 (Omicron)	Th1	(30)
CuMV	Genetic fusion	RBM	——	94 nm	Subcutaneous inoculation	2 doses (20 µg, 28 days apart) 2 doses (100 µg, 28 days apart)	Mice	——	SARS‐CoV-2/ABS/NL20	——	——	(32)
Ferritin	SpyTag/SpyCatcher	RBD and HR	RBD:319-541 HR1-HR2:910-1213	——	Subcutaneous inoculation	2 doses (10 µg, 28 days apart)	Mice	WT, SARS-CoV, MERS-CoV, HCoV-229E, HCoV-OC43, RATG13	SARS-CoV-2	10-100	Th1	(35)
					Intramuscular injection	2 doses (50 µg, 28 days apart)	Rhesus macaques					
	Genetic fusion	Spike	12-1158 from the WT	——	Intramuscular injection	2 doses (50 µg, 28 days apart)	Rhesus macaques	WT	SARS-CoV-2 viruses USA-WA1/2020 (WA1), Alpha, Beta, Gamma, and Delta	——	Th1	(36)
	Protein A tag-Fc	RBD	331-524 from omicron BA.1	37.3 ± 1.3 nm	Intramuscular injection	2 doses (10 µg, 14 days apart)	Mice	WT, Alpha, Beta, Gamma, Delta, Omicron BA.1, BA.2	BA.1	——	Th1	(37)
	Genetic fusion	Spike	1-1208 from the WT	——	Intramuscular injection	1 dose (100 µg)	Hamsters	WT, D614G, Alpha, Beta, Delta	SARS-CoV-2 WA1/2020 strain	——	——	(38)
	Genetic fusion	RBD (SARS-CoV, SARS-CoV-2, MERS-CoV),	——	17.6 nm	Intramuscular injection	2 doses (10 µg, 21 days apart)	Mice	——	SARS-CoV, MERS-CoV, SARS-CoV-2	——	Th1	(41)
						2 doses (100 µg, 21 days apart)	Cynomolgus monkeys					
	Genetic fusion	Spike (WT, Beta, Epsilon)	——	21.4 nm, 20.54 nm, 20.3 nm	Subcutaneous inoculation	2 doses (50 µg, 23 days apart)	Mice	——	——	——	Th1	(59)
	ΔN1-SpyCatcher	RBD	319–541 from the WT	32.99 ± 0.04 nm	Subcutaneous inoculation	3 doses (9.34 µg, 14 days apart)	Mice	WT	SARS-CoV-2 strain 2020XN4276	8-120	Th2	(48)
	SpyTag/SpyCatcher	S1	16-685 from the WT	57.71 ± 0.22 nm	——	3 doses (0.646/6.46 µg, 14 days apart)	Mice	WT, Alpha, Beta, Gamma, Delta, Lambda, and Omicron, SARS-CoV, MERS-CoV	SARS-CoV-2 strain 2020XN4276	30	——	(49)
	GvTagOpti/SdCatcher	D614G_RBD	——	——	Subcutaneous inoculation	2 doses (12 µg, 28 days apart)	Mice	D614G, Alpha, Beta, Gamma, Delta, Omicron	D614G, Beta, Delta	——	——	(57)
Mi3	SpyTag/SpyCatcher003	RBD	331–529	20.7 ± 4.2 nm	Intramuscular injection	2 doses (0.1/0.5 µg, 14 days apart)	Mice	SARS-CoV-2	SARS-CoV-2 virus (hCoV-19/England/02/2020)	——	——	(45)
						2 doses (5/50 µg, 28 days apart)	Pigs					
	ΔN1-SpyCatcher	RBD	319–541 from the WT	55.19 ± 0.4	Subcutaneous inoculation	3 doses (9.51 µg, 14 days apart)	Mice	WT	SARS-CoV-2 strain 2020XN4276	8-120	Th2	(48)
	SpyTag/SpyCatcher	S1	16-685 from the WT	65.88 ± 0.69 nm	——	3 doses (6.46 µg, 14 days apart)	Mice	WT, Alpha, Beta, Gamma, Delta, Lambda, Omicron, SARS-CoV, MERS-CoV	SARS-CoV-2 strain 2020XN4276	8	——	(49)
I53–50	Genetic fusion	RBD	328-531	37-41 nm	Intramuscular injection	2 doses (0.9/5 µg, 21 days apart)	Mice	SARS-CoV-2, SARS-CoV	SARS-CoV-2	——	——	(47)
						2 doses (250 µg, 28 days apart)	Pigtail macaque					
	ΔN1-SpyCatcher	RBD	319–541 from the WT	50.67 ± 0.11 nm	Subcutaneous inoculation	3 doses (11.91 µg, 14 days apart)	Mice	WT	SARS-CoV-2 strain 2020XN4276	8-120	Th2	(48)
	SpyTag/SpyCatcher	S1	16-685 from the WT	90.00 ± 0.27 nm	——	3 doses (0.721/7.21 µg, 14 days apart)	Mice	WT, Alpha, Beta, Gamma, Delta, Lambda, Omicron, SARS-CoV, MERS-CoV	SARS-CoV-2 strain 2020XN4276	39	——	(49)
PPS14	Chemically conjugated	RBD	319–531	8-23 nm	Intramuscular injection	3 doses (1/3/10 µg, 21 days apart)	Adult mice	WT, D614G, Alpha, Beta, Gamma, Delta, Omicron, Lambda, Mu, Epsilon, Iota, and Kappa	——	3.7	Th1 (mice), balanced Th1 and Th2 (rhesus macaques)	(51)
						3 doses (1/3 µg, 21 days apart)	Aged mice					
						3 doses (20/60 µg, 21 days apart)	Rats					
						2 doses (10 µg, 21 days apart)	Rhesus macaques					
PRBS	Hydrogen bond	RBD	——	250 nm	Intramuscular injection	3 doses (10 μg RBD protein and 50 μg PRBS, 14 days apart)	Mice	SARS-CoV-2	——	5	——	(52)
DNA	Plasmids	S-HBsAg	1-1206 from the S6P	——	Intramuscular immunizations plus electroporation	2 doses (10/2/0.4 µg, 28 days apart)	Mice	D614G, Beta, Delta, Omicron BA.1	——	3-26.5	——	(54)
	Plasmids	RBD g5.1 120-mer	331-527	——	Intramuscular immunizations plus electroporation	1 dose (1 µg)	Mice	Alpha, Beta, Gamma, Delta	SARS-CoV-2	15	——	(55)
RNA	LNP encapsulation	RBD-ferritin	——	121.9 nm	Intramuscular injection	2 doses (1.5/15 µg, 14 days apart)	Mice	WT, Alpha, Beta	SARS-CoV-2	——	Th1	(56)

### 2.1 VLPs

VLPs are virus-like man-made nanostructures consisting of all or part of the proteins that make up the viral capsid, but lacking the viral genome (21). These structural proteins can self-assemble when produced in expression organisms such as bacteria, yeast, mammalian, and insect cells (22). However, not all pathogenic proteins can self-assemble into VLPs after overexpression, and 110 viral proteins in 35 virus families can self-assemble (23, 24).

#### 2.1.1 Phage

MS2 phage is an icosahedral structure of 180 monomeric coat proteins self-assembling to form 90 homodimers (20) (**Figure 1A**). The AB loop in the homodimer is exposed on the surface and is a natural site for delivery of antigen (25). Genomic insertion of exogenous antigens into the AB loop tends to cause protein folding failure, a defect that can be ameliorated by constructing single-stranded homodimers, which greatly improves protein stability (25). Based on this research, AviTag was inserted into the single-stranded dimer and Spike (S) for biotinylation, then S was displayed on the surface of MS2 in the presence of high-affinity biotin-streptavidin. The vaccine provided protection against SARS-CoV-2 after a single injection in hamsters (26).

#### 2.1.2 Plant viruses

Most plant viruses are envelope-free and their viral particles are formed by highly repetitive protein subunits assembled around the genome, which are basically divided into two symmetrical types of structure: helical and icosahedral (27). The most common helical plant virus is tobacco mosaic virus (TMV) (**Figure 1B**). Since WT TMV lacks exposed active lysine, Royal et al. mutated the N-terminal end of TMV with lysine (TMV NtK) to facilitate the chemical coupling of the SARS-CoV-2 receptor-binding domain (RBD)-Fc with it to construct a vaccine that triggered IgG antibody titers more than 10-fold higher than the RBD alone (28). The adjuvanted nanoparticle vaccine triggered more Th1-oriented cellular immunity, while the non-adjuvanted nanoparticle vaccine resulted in a more balanced Th1 and Th2 response (28). Free monomeric antigens stimulate mainly Th2 responses compared to nanoparticle vaccines (28). Papaya mosaic virus (PapMV) vaccine platforms are rod-like virus-like particles made of PapMV capsid protein self-assembled around single-stranded RNA (ssRNA) (29). PapMV nanoparticles are one of the few vaccine technologies capable of stimulating TLR7/8, other TLR7/8 agonists are not suitable for developing safe vaccines due to their toxicity (30). SRAS-CoV-2 S RBD coupled to PapMV by relying on sortase A, which can trigger the creation of covalent junctions between proteins (30). The sera produced after two immunizations of mice were 54, 111, and 5.2 times more efficient in neutralizing the ancestral SARS-CoV-2 strain, Delta and Omicron variant, respectively, than the uncoupled formulation (30). The RBD-PapMV vaccine can be stored stably at 4 ± 3°C for one month, which is an advantage over mRNA vaccines that require refrigeration (30). The generic T-cell epitopes derived from tetanus toxin was incorporated into the cucumber mosaic virus (CuMV) VLP platform (CuMV_TT^–^_VLP) for immune optimization to obtain T-cell help (31). The vaccine constructed by chemically coupling the RBD to CuMV_TT_-VLP prevents the binding of the RBD to the ACE2 receptor, and in addition, the antibodies induced in mice are effective in neutralizing SARS-CoV-2 (31). Delivery of oversized or positively charged proteins on the outer surface of plant viruses by genetic fusion can affect the yield of plant viruses (27). To improve yields, genetically fused smaller fragments of receptor-binding motif (RBM) was displayed into CuMV_TT_ to construct the vaccine candidates (32). Sera from mice vaccinated with the CuMV_TT_-RBM vaccine candidate successfully recognized variants E484K, N501Y, K417N/E484K/N501Y and L452R/E484Q (32).

### 2.2 SANPs based on the ferritin system

Ferritin is one of the oldest molecule that is found in a variety of biological species such as fungi, archaea, bacteria, and viruses. Ferritin has excellent thermal and chemical stability and vaccines made upon it are useful in countries and regions with limited cold chain supply resources (23, 33). Ferritin is an octahedral nanoparticle consisting of eight identical trimeric subunits and therefore can display 24 copies of RBD or 8 copies of stable S-protein trimers on a single particle (8, 34) (**Figure 1C**). Simultaneously expression of SRAS-CoV-2 S RBD and heptad repeat 1(HR) on a single ferritin nanoparticle induced a 10-100-fold higher neutralizing potency than the monomeric antigens, as well as a safer and more effective Th1-based immune response (35). In mice injected with ferritin nanoparticle vaccines and monomeric antigens, respectively, and lymph nodes (LNs) were isolated for analysis of DCs and macrophages, the nanoparticle vaccine was found to be more susceptible to antigen capture and presentation by APCs, which in turn effectively activated CD8 T cells (35). Utilizing gene fusions, the prefusion-stabilized S has been expressed on the surface of ferritin to induce immune responses against SARS-CoV-2 (36). The vaccine triggered high levels of neutralizing antibodies in rhesus monkeys, an order of magnitude higher than human recovered serum, and induced Th1-biased CD4 T-cell helper responses (36). Serum from mice, immunized with Omicron RBD linked to ferritin by Fc interaction with protein A tags, showed strong neutralizing potency against BA.1 and BA.2 and Th1-biased cellular immune responses (37). Attached the WT strain S protein to ferritin by gene fusion and the nanoparticle vaccine demonstrated similar protection against WT, D614G, and Alpha in Syrian golden hamsters (38). A recombinant spike ferritin nanoparticle vaccine for SARS-CoV-2 has entered human clinical trials for the first time, demonstrating the ability to elicit neutralizing antibody titers exceeding 10,000 against the D614G variant in humans. This vaccine primarily causes self-limiting adverse reactions and shows no evidence of long-term toxicity (39). Additionally, conserved epitopes of pre-existing neutralizing antibodies (CePn) were delivered using *Helicobacter pylori* ferritin to generate nanoparticle vaccines that protect mice against the Delta, WIV04, and Omicron variants (40). Ferritin nanoparticle vaccines that present the RBD of SARS-CoV, MERS-CoV, and SARS-CoV-2 simultaneously elicited Th1-biased immune responses in mice, providing protection against all three types of β-coronaviruses (41).

### 2.3 Engineering self-assembling protein

The naturally occurring self-assembling proteins described above are generally chosen as the platform for antigen delivery, however, the number of naturally occurring self-assembling proteins is rather limited (8). With advances in bioengineering, it is currently possible to artificially modify the size of the nanoparticles, the spacing, and the number of antigens presented to further optimize the next generation of vaccines (8). Computationally constructed I53-50 and mi3 (the variant of I3-01) self-assembled nanoparticles have been successfully applied in SARS-CoV-2 vaccine design (42, 43). The mi3 is a dodecahedral one-component nanocage that can deliver 60 monomers on the surface (44) (**Figure 1D**). Construction of nanoparticle vaccine based on SpyTag/SpyCatcher technology for delivery of RBD on SpyCatcher003-mi3 platform induces stronger immune responses in mice and pigs compared to human recovered serum (45). Patel et al. developed an intranasal nanoparticle vaccine with modified the I3-01 protein into nanoparticles with a flexible SpyCatcher system to display the RBD. On day 5 after infection, the number of virus particles in golden Syrian hamsters vaccinated with the nanoparticle vaccine was 10 times lower than in those vaccinated with the monomer vaccine. Nanoparticle vaccine cleared viral particles from the respiratory tract much faster than the monomer vaccine (46). I53-50 is an icosahedral symmetric protein oligomer composed of 20 trimers (I53-50A) and 12 pentamers (I53-50B) (34, 43) (**Figure 1E**). The RBDs were linked to I53-50A by genetic fusion, and RBD-I53-50A and I53-50B were expressed independently and then mixed and assembled into nanoparticle vaccine (47). The vaccine induced a 10-fold higher neutralizing activity in mice at lower doses than the pre-fused stable S protein and a robust generating center (GC) B-cell response, favoring a durable humoral response (47). The ΔN1-SpyCatcher system was designed to express SARS-CoV-2 RBD on ferritin, mi3, and I53-50 NPs to construct nanoparticle vaccines, and the sera obtained after immunization of mice with the vaccines showed 8 to 120 times higher neutralizing activity against pseudoviruses and real viruses than sera from mice immunized with monomeric RBD (48). Three different sizes of nanoparticle vaccines were constructed by linking WT S1 to ferritin, mi3, and I50-53 via SpyTag/SpyCatcher system (49). Among them, the I50-53 nanoparticle vaccine induced the highest neutralizing antibody titer in mice, which was 39 times higher than that of the S1 monomeric vaccine, while the mi3 nanoparticle vaccine had the lowest neutralizing potency, which was only 8 times that of the S1 monomeric vaccine (49). The number of antigens on the surface of the particles does not necessarily correlate positively with the neutralizing antibody titer. Overall, all three vaccines produced high levels of neutralizing antibodies against multiple variants of SARS-CoV-2, including the Omicron variant, and protection against SARS-CoV and MERS-CoV (49).

### 2.4 Natural polysaccharides

Similar to the protein-based SANPs described above, natural polysaccharides can also be assembled through non-covalent interactions (50). A polysaccharide-protein conjugated nanoparticle vaccine against SARS-CoV-2 was constructed by coupling the SARS-CoV-2 recombinant RBD protein to capsular polysaccharide of streptococcus pneumoniae serotype 14 (PPS14) using a reductive amination method (51). This SANP triggers 3.7-fold higher neutralizing antibodies than the monomer RBDs vaccine and induces a Th1-biased immune response in mice while inducing a balanced Th1 and Th2 immune response in rhesus monkeys (51). A new natural polysaccharide was extracted from Bletilla striatal rhizomes (PRBS), which can be used for the development of a SARS-CoV-2 vaccine (52). Compared to the RBD monomeric vaccine, the PRBS-RBD vaccine triggered 5-fold higher neutralizing antibodies in mice and promoted phagocytosis of phagocytes and antigen presentation by B cells (52).

## 3 Nucleic acid-encoded nanoparticle vaccine platforms assembled *in vivo*

The production process of SANPs vaccines is mainly prepared *in vitro*, using protein expression systems and protein purification techniques, which can be costly. The incorporation of components like SpyTag/SpyCatcher necessitates even more expensive purification methods, such as size exclusion chromatography. In the event of an outbreak, these cumbersome preparation steps and high costs may hinder the rapid development and scaling of vaccines. Therefore, the *in vivo* assembly of self-assembled nanoparticle vaccines could represent a new paradigm in this field (53).

DNA and RNA vaccines are two approaches that enable *in vivo* self-assembly. Antigens encoded in DNA or RNA can enter host cells through methods like electroporation, jet delivery, or encapsulation in nanoparticles. Once inside, nanoparticle proteins are expressed and successfully assembled *in vivo*. For instance, Liu et al. designed a DNA plasmid encoding a Spike-HBsAg fusion protein, which was delivered to mice via intramuscular injection combined with electroporation. 2 µg DNA vaccine provided protection for over 7 months and exhibited higher neutralization activity compared to equal doses of soluble protein (54). Similarly, Konrath et al. discovered that DNA vaccines encoding nanoparticle proteins maintained high immunogenicity; a single dose of just 1 µg of a DNA-launched RBD g5.1 120-mer nanovaccine was sufficient to protect mice from SARS-CoV-2 challenges (55). Sun et al. prepared mRNA encoding RBD-ferritin nanoparticles, encapsulated in LNP, to induce a Th1-biased immune response in mice (56). These LNPs can simultaneously encapsulate three mRNAs corresponding to different mutant strains (WT, Alpha, Beta) of RBD, resulting in a trivalent vaccine that generates broad-spectrum neutralizing titers against a variety of pseudovirus (56).

## 4 Developing broad-spectrum vaccines with nanoparticle platforms

The RBD in S is a hot region of constant mutation and vaccines made using the WT S have shown varying degrees of reduced protection against multiple variants such as Alpha, Beta, Gamma, Delta, Lambda, and Omicron (38, 49). Thus, one of the biggest hurdles for a SARS-CoV-2 vaccine is the development of a universal vaccine that can overcome the viral immune escape and maintain neutralizing efficacy against multiple variants of concern.

**Figure 2:**
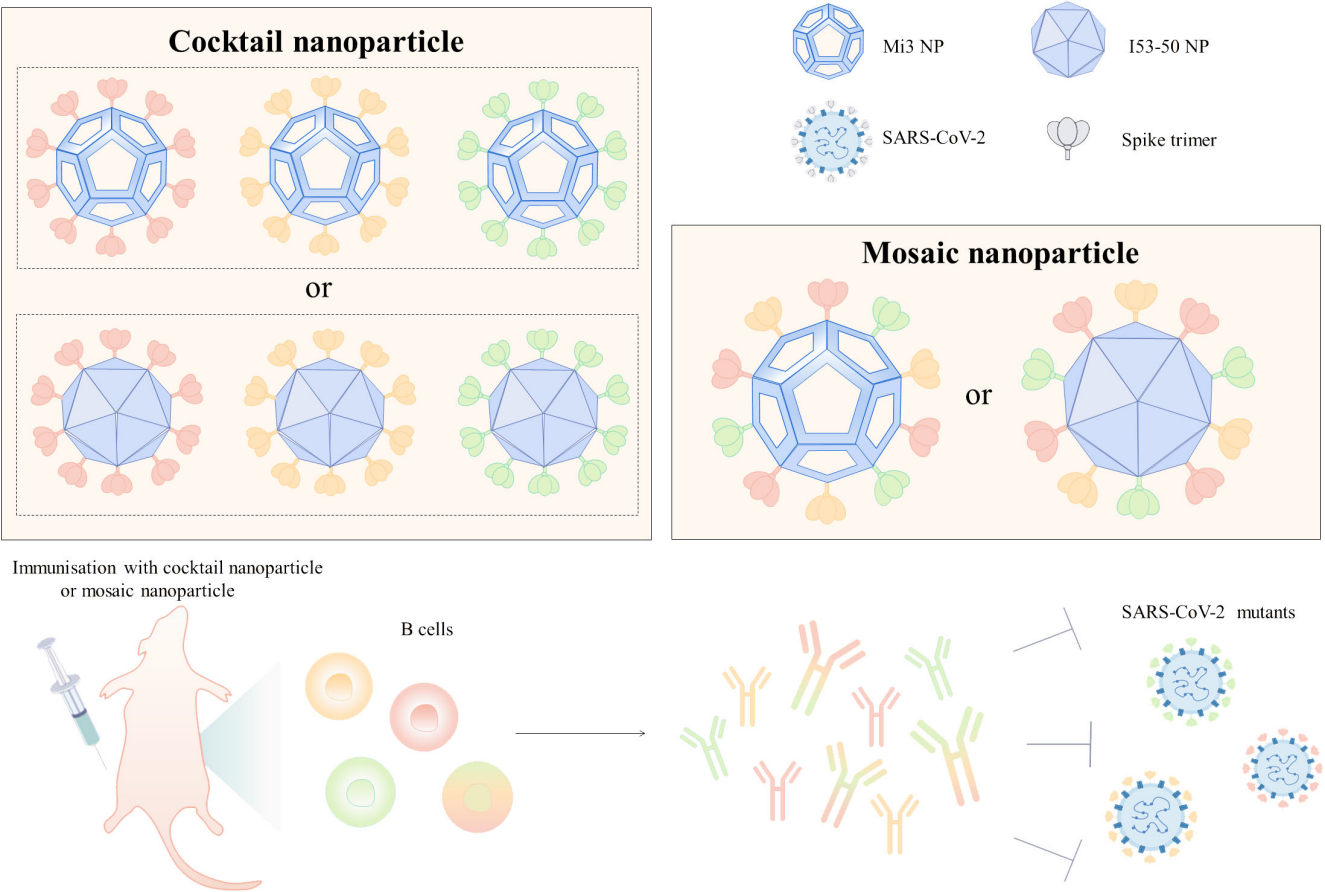
Design of broad-spectrum vaccines based on nanoparticle platforms. Cocktail or mosaic nanoparticle vaccines are commonly used strategies for broad-spectrum vaccine design, and Mi3 and I53-50 are mostly used for broad-spectrum vaccine design because of the high number of antigens that can be delivered on their surfaces. Mice injected with cocktail or mosaic nanoparticle vaccines developed resistance to infection with multiple SARS-CoV-2 mutant strains. In the figure, the different colored S trimers are from different strains.

Cocktail nanoparticle vaccines and mosaic nanoparticle vaccines are capable of inducing a wider range of neutralizing antibodies and are commonly used to construct universal vaccines against SARS-CoV-2. Cocktail nanoparticles are combinations of three or more independently assembled nanoparticles expressing only a single type of antigen (**Figure 2**). For instance, the mixing of three ferritin nanoparticle vaccines linked to D614G, Beta, and Delta RBD in a 1:1:1 ratio forms a cocktail of nanoparticle vaccines (57). There were no pathological changes in the lungs and the viral RNA was almost undetectable in mice injected with this trivalent vaccine after infection with the SARS-CoV-2 variant (57). Co-injection of D614G and Beta ferritin nanoparticle vaccines as booster shots into rhesus monkeys effectively induced antibodies to neutralize multiple variants of interest, including D614G, Alpha, Beta, Gamma, and Delta, and increased the neutralization potency by 21-110-fold (58). Three different RBD antigens derived from WT, Beta, and Epsilon were used to construct three ferritin nanoparticle vaccines utilizing genetic fusion, respectively (59). In mice immunized with monovalent and polyvalent vaccines, respectively, the number of memory cells in the LN was higher in the mice of the polyvalent vaccine group (59). In addition, mosaic nanoparticle vaccines can bind multiple antigens on the surface of a single nanoparticle, where the nanoparticles were mostly selected from I53-50 or mi3 (**Figure 2**). A quadrivalent mosaic nanoparticle vaccine was constructed by co-associating S of WT, Alpha, Beta, and Gamma with I50-53, which improved the breadth of neutralizing antibodies and showed 1.6-4 times higher neutralizing antibody titers against several variants of SARS-CoV-2 (Alpha, Beta, Gamma, Omicron, Lambda, Eta, and D614G) than the WT monovalent nanoparticle vaccine (60). Bivalent nanoparticle vaccines co-expressing WT and Beta S on I53-50 nanoparticles triggered 6.2, 4.7, 7.3, and 4.6-fold higher neutralizing antibody titers against Beta, Gamma, and Omicron BA.1 and BA.4/5, respectively, in rabbits than WT monovalent nanoparticle vaccines (61).

The strategy of the mosaic nanoparticle vaccine is also applicable to the development of a universal vaccine against sarbecovirus. Bivalent mosaic nanoparticle vaccines co-expressing S constructs of SARS-CoV and SARS-CoV-2 on the surface of I53-50 nanoparticles produce potent and high-level neutralizing antibodies against SARS-CoV, SHC014, and WIV1 (61). An 8-valent mosaic mi3 nanoparticle vaccine expressing eight different zoonotic coronavirus RBDs simultaneously increased the breadth of antibody recognition of heterologous RBDs compared to homologous SARS-CoV-2 RBD nanoparticle vaccines (62). Similarly, an octavalent mosaic mi3 vaccine co-expressing SARS-CoV-2 Beta and seven animal sarbecoviruses RBDs increased targeting of conserved epitopes and induced antibodies binding mismatched viruses such as WA1, Delta, and Omicron RBDs in non-human primates, offering the possibility of preventing future sarbecoviruses -induced diseases (63). The mosaic-8 nanoparticles are currently undergoing clinical trials. However, the large-scale production of this type of vaccine is constrained by the need to produce nine components (eight different receptor-binding domains, or RBDs, along with SpyCatcher003-mi3). To address this challenge, Hills et al. have introduced an innovative solution by developing multiviral quartet nanocages. In this approach, four viral RBDs (SHC014, Rs4081, RaTG13, and SARS-CoV-2) are linked into a single peptide chain. This chain is then assembled into SpyCatcher003-mi3 using SpyTag, resulting in a nanoparticle vaccine with a branching morphology that elicits a broad immune response against sarbecoviruses (64). The development of broad-spectrum vaccines utilizing mosaic nanoparticles should prioritize the spatial arrangement and proportions of heterotypic antigens. Simply increasing the number of antigens without careful consideration can lead to a reduction in neutralization potency and may trigger excessive inflammatory responses (65).

## 5 Nanoparticle vaccine activates immune cells - APCs, B cells, and T cells

**Figure 3:**
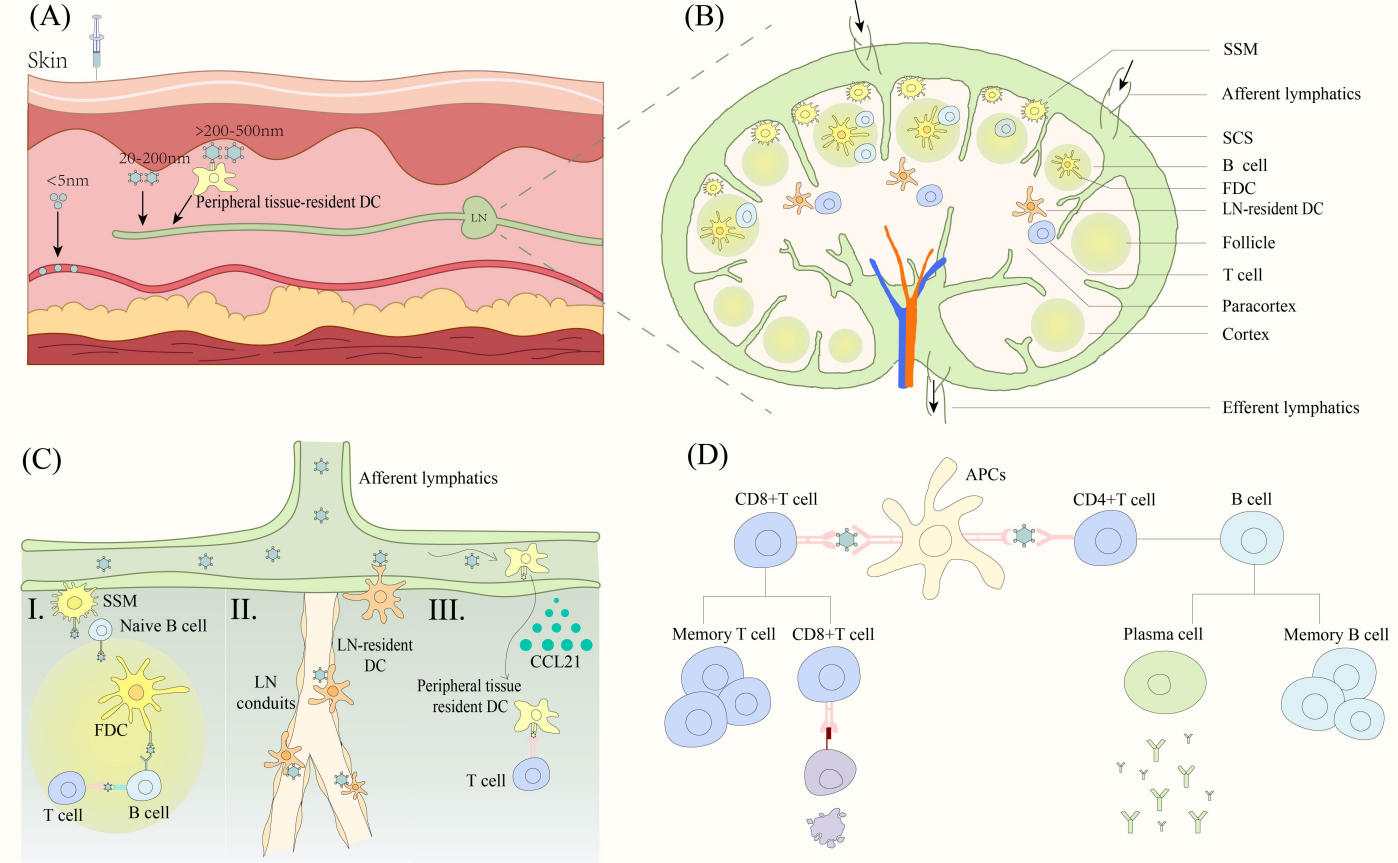
Nanoparticle vaccine triggers the immune response. **(A)** Nanoparticles of different sizes enter the LN. Particles smaller than 5 nm mainly enter capillaries. 20-200 nm particles drain directly into LNs. Particles larger than 200-500 nm enter the LNs by active transport of DC cells. **(B)** Structure of LN and cellular distribution. **(C)** Nanoparticles contacted with different APCs. (I) Antigen is presented to B cells via SSM, FDC to trigger an adaptive immune response. (II) LN resident DCs capture antigen at two sites. Antigens are captured by DCs near the lymphatic endothelium after entering the SSM or by DCs near the duct after entering the lymphatic conduit. (III) DCs that have captured antigen in peripheral tissues migrate to the paracortical region of LNs in response to low to high concentrations of CCL21, presenting the antigen to T cells to trigger an adaptive immune response. **(D)** APC presents antigens to T cells and B cells to trigger a cellular and humoral immune response respectively. DC, dendritic cells. LN, lymph nodes. SSM, subcapsular sinus macrophage. SCS, subcapsular sinus. FDC, follicular dendritic cells. APCs, antigen presenting cells.

Vaccine antigens primarily activate the body’s adaptive immune system to fight off the invasion of foreign pathogens. The LNs are the primary site for the initiation of the adaptive immune response and therefore antigens first need to be transported from the injection site into the LN. Nanoparticles of different sizes enter the LN through different pathways after injection into the interstitium (66) (**Figure 3A**). Particles that are too small (<5nm) enter the capillaries because they diffuse faster than convection and vaccination is ineffective because of clearance effects (66, 67). Particles between 20 and 200 nm are effective in entering the lymphatic system (68), with medium-sized particles (20 to 50 nm) being the best size as they are more likely to be convectively governed to enter the lymphatic vessels (66). Larger particles above 50 nm slow down the rate of entry into the lymph vessels as they increase in size (66). Most protein-based SANPs and VLPs are concentrated in the 20-200 nm size range and are suitable for direct LN drainage. Particles over 200-500 nm become trapped in the interstitial space, increasing the chance of contact with APCs such as DCs in peripheral tissues and transport to lymph vessels via APCs (69).

The nanoparticles entering the lymphatic vessels reach the LN with the lymphatic fluid. The particles are distributed in the subcapsular sinus (SCS) and over time penetrate deeper into the lymphatic parenchyma such as the cortical and paracortical areas (69). The SCS is rich in macrophages and below it is the paracortical area with T cells and resident DCs as well as the cortical area with B cells (70, 71) (**Figure 3B**). The nanoparticles in turn trigger T and B cell responses upon contact with lymphatically resident APCs. However, the exact pathway of movement of nanoparticles deep into the LN parenchyma is not fully understood.

Deeper transfer of particles from the SCS and around the B-cell follicle to the interior of the B-cell follicle can be observed in the experiments of Manolova et al. (69). This process may be mediated by subcapsular sinus macrophages (SSM) (**Figure 3C**). SSM transports antigen to naive B cells, which transport it from the SCS to the follicle and the follicular dendritic cells (FDCs) in a complement receptor-dependent manner (72). FDCs endocytose antigen and present it to GC B cells, which are activated and differentiated into antibody-producing plasma cells or memory cells with the help of T cells (72). It is also possible that nanoparticles can penetrate from the lymphatic ducts deep into the T cell zone of the LN (**Figure 3C**). It is often thought that particles larger than 4-5 nm are excluded from the conduits (73), however, this is not absolute and cowpox and Zika particles have been shown to penetrate deeply into the parenchyma through the lymphatics (74). LN-resident DCs located near the LN conduits capture and present antigen to the T cells (74). In addition, there is a specialized population of DCs residing in the lymphatic endothelium, capable of capturing granular antigens from the lymphatic sinus (75) (**Figure 3C**). Finally, a few particles are possibly captured by peripheral tissue-resident DCs (69) (**Figure 3C**). Lymphatic endothelial cells secrete CCL21 chemokine, and CCRL1 can bind and remove CCL21 from the lumen of the lymphatic sinus, thus creating a gradient of high to low CCL21 concentrations from the LN parenchyma (inside the LN) to the subepithelial lymphatic sinus (LN margin), and peripheral tissue-resident DCs migrate to the paracortical layer by upregulating the chemokine receptor CCR7, which in turn presents antigen to surrounding T cells (76, 77).

Based on all these pathways, nanoparticle vaccines are exposed to various APCs and are phagocytosed into the endosome (66). CD8+T cells recognize the antigens presented by major histocompatibility complex (MHC) class I molecules through the T cell receptor (TCR), which are activated in the presence of co-stimulatory factors and cytokines, and subsequently activated CD8+T cells induce cytotoxicity to kill the virus-infected cells (78). CD4 helper T cells are activated by TCR recognition of peptides binding to MHC class II molecules on the surface of DCs (78). Activated CD4 helper T cells differentiate into memory T cells that play a role in secondary immunity and follicular T cells that secrete molecules such as IL-21 to aid B cell differentiation (79). B cells with the help of T cells differentiate into memory cells as well as plasma cells, which produce antibodies to bind to the virus (80) (**Figure 3D**).

## 6 Conclusion and perspective

Nanoparticle vaccines have great potential in vaccine development, where multiple vectors are applied, each with their own characteristics, to address the limitations of current vaccine technology. The diversity of VLP structures and their expression hosts, which are upwards of 170, make them attractive. The strong thermal and chemical stability of ferritin makes it ideal for carrying antigens. Certain modifications of proteins by computational design can further improve the stability or yield of nanoparticles. Engineering SANPs also favored the design of universal vaccines. Bacterial polysaccharides vaccines are well developed and the coupling of viral proteins to polysaccharides can prevent bacterial and viral co-infection. Nucleic acid-emitting nanovaccines offer a promising new approach that enables the *in vivo* synthesis of self-assembled proteins. This method helps to overcome the manufacturing and cost challenges typically associated with *in vitro* production. In future, the emergence of new viruses is unpredictable, and accelerating the development of nanoparticle vaccines could shorten the response time to unknown epidemics. Although there is no doubt that nanoparticle vaccines can improve immunogenicity, we should still work on continuous development and optimization of nanoparticle vaccines, such as improving the delivery efficiency of the carriers and optimizing the distribution and quantity of antigens. In addition, the deployment of viral vaccines still varies globally, and vaccine cost and storage are issues that need to be urgently addressed. Therefore, it is necessary to develop more stable and easier-to-produce nanoparticle vaccines. In conclusion, nanoparticle vaccines are an effective way to combat infectious viruses, and it is believed that they can be used to improve the health of all human beings in the near future.
